# Store-Operated Calcium Channels as Drug Target in Gastroesophageal Cancers

**DOI:** 10.3389/fphar.2021.668730

**Published:** 2021-05-03

**Authors:** Yan Chang, Souvik Roy, Zui Pan

**Affiliations:** ^1^ College of Nursing and Health Innovation, The University of Texas at Arlington, Arlington, TX, United States; ^2^ Department of Mathematics, The University of Texas at Arlington, Arlington, TX, United States

**Keywords:** orai, esophageal squamous cell carcinoma, drug resistance, cancer stem cells, calcium signaling

## Abstract

Gastroesophageal cancers, including tumors occurring in esophagus and stomach, usually have poor prognosis and lack effective chemotherapeutic drugs for treatment. The association between dysregulated store-operated calcium entry (SOCE), a key intracellular Ca^2+^ signaling pathway and gastroesophageal cancers are emerging. This review summarizes the recent advances in understanding the contribution of SOCE-mediated intracellular Ca^2+^ signaling to gastroesophageal cancers. It assesses the pathophysiological role of each component in SOCE machinery, such as Orais and STIMs in the cancer cell proliferation, migration, and invasion as well as stemness maintenance. Lastly, it discusses efforts towards development of more specific and potent SOCE inhibitors, which may be a new set of chemotherapeutic drugs appearing at the horizon, to provide either targeted therapy or adjuvant treatment to overcome drug resistance for gastroesophageal cancers.

## Introduction

### Gastroesophageal Cancers

Gastroesophageal (GE) cancers are malignancies that occur in upper gastrointestinal track, including esophageal, gastric and gastroesophageal junction, and are usually presented with poor prognosis (Hsu et al., 2020). Gastric carcinoma (GC) is the fourth leading cause of death of all cancers in the world with 5°year survival rate of 10–30% (Jemal et al., 2011; Sung et al., 2021). For esophageal cancer (EC), the numbers of new case and death in 2020 were 604,000 and 544,000, respectively (Sung et al., 2021). EC is the sixth leading cause of cancer death with 5°year survival rate less than 20% (Zhang and Pan, 2020).

Histologic classification identifies two major types of GC: intestinal and diffuse types (Lauren 1965). The intestinal type of GC is believed to be affected by environmental factors such as *H. pylori* infection in old ages (Warren et al., 1983; Zhang and Pan, 2020). The diffuse type is highly associated with Epstein-Barr virus (EBV) infection and specific genetic alterations such as *CDH1* in young ages (Henson et al., 2004). Other factors also contribute to GC that include smoking (Ladeiras-Lopes et al., 2008), alcohol consuming (Jedrychowski et al., 1986) and obesity (Vaughan et al., 1995). EC histologically also has two most common types, i.e. esophageal adenocarcinoma (EAC) and esophageal squamous cell carcinoma (ESCC). ESCC arises from the lining epithelial cells in the upper part of esophagus; and EAC arises from glandular cells present in the lower third of the esophagus, often occurring at transformed Barrett’s esophagus (di Pietro et al., 2018; Wheeler and Reed, 2012). The risk factors for ESCC include alcohol consumption (Brooks et al., 2009), smoking (Morita et al., 1994), dietary zinc deficiency (Choi et al., 2018), and mechanical insults (Lambert and Hainaut, 2007).

### Current Treatment for Gastroesophageal Cancers

Symptoms of GE include dyspepsia, early satiety, pain, and symptoms of anemia (Sehdev and Catenacci, 2013). Besides biopsy pathology evaluation, endoscopic ultrasound and computerized tomography (CT) scan or positron emission tomography (PET) scan of chest, abdomen, and pelvis are employed in the diagnosis of GE cancer. Surgery, radiotherapy and chemotherapy are the main treatments for GE cancers. The current chemotherapy drugs include cisplatin, 5-fluorouracil (5-FU), paclitaxel, or the combination (Jin et al., 2004). The standard curative intent treatment for both ESCC and EAC is 5–6 weeks of neoadjuvant chemoradiation (CROSS), a combination of paclitaxel and carboplatin with a cumulative radiation dose of 41.4 Gy over 23 fractions followed by esophagectomy (van Heijl et al., 2008). Of course, these standard treatments lead to permanent damage to organs, significant side effects and impede life quality of patients that recover from GE cancers.

### Targeted Therapy for Gastroesophageal Cancers

Targeting therapy has been attracted attention in past few years for its benefit of less side-effects than conventional treatment. Targeting epidermal growth factor receptor (EGFR) family of receptor tyrosine kinases (RTKs) have been approved as a successful approach for lung, breast and other cancers. EGFR/RTK has four members including EGFR (HER1), HER2, HER3, and HER4. EGFR is a 170 kDa transmembrane receptor on cell membrane. Upon activation, EGFR triggers activation of MAPK, STAT5, and Ras-Raf-MEK pathways resulting in a cascade signaling of cell proliferation and survival (Yano et al., 2003). As a matter of fact, EGFR/HER signaling pathways regulate almost all aspects in cancer biology including cell growth, survival, adhesion, migration, and differentiation. Different from mutant forms in lung cancer and breast cancer, EGFR often presents high copy number and its expression is correlate with advanced stage, poorly differentiated histology, vascular invasion, and poor survival rate in GC and EC (Ku and Ilson, 2013; Wang et al., 2016) (Terashima et al., 2012). Additionally, HER2 is often overexpressed together with EGFR in a significant amount of EC patients as well.

Inhibiting EGFR/RTKs pathways can be achieved either by monoclonal antibody (mAb) or downstream by tyrosine kinase inhibitor (TKI). Several mAbs and more than 20 TKIs have been approved by FDA. For example, cetuximab and panitumumab specifically bind EGFR and trastuzumab and pertuzumab target HER2. Afatinib is a pan-HER family TKI, but selectively and irreversibly inhibits EGFR, HER2, and HER4, and blocks transphosphorylation of HER3. While many of these mAbs and TKIs have been approved by FDA for treatment of lung, colon, breast, or head and neck cancers, their therapeutic benefits for EC are still unclear (Jiao et al., 2018). For example, trastuzumab, a monoclonal antibody against HER2, is the only FDA approved EGFR targeting treatment for EC but has limited response rate (Kurokawa et al., 2014; Doi et al., 2017; Yang et al., 2020). It has been used in combination with 5-FU or cisplatin for HER2 positive GC (Bang et al., 2010). Ramucirumab, a monoclonal antibody of vascular endothelial growth factor receptor-2 (vEGFR-2), was used with paclitaxel together in GC treatment (Wilke et al., 2014). Other agents such as afatinib and bevacizumab, are still in clinical trials (Spicer et al., 2017). Despite the high expectation of TKIs in GC and EC therapy, many clinical trials of TKIs either alone or combined with other therapies have produced disappointing results to date (Wang et al., 2016) and the 5 year survival rate of EC patients is still below 20% (Siegel et al., 2017). No clinical benefit has been harvested in phase III trials with cetuximab (EXPAND), panitumumab (REAL3), and gefitinib (COG) (Maron et al., 2020). In EXPAND, capecitabine and cisplatin, with or without cetuximab, are used for patients with previously untreated advanced gastric cancer (Lordick et al., 2013). In REAL3, epirubicin, oxaliplatin, and capecitabine, with or without panitumumab, are used in patients with previously untreated advanced GE cancer (Waddell et al., 2013). In COG, gefitinib has been evaluated in esophageal cancer patients after chemotherapy (Dutton et al., 2014).

The failure in all above mentioned Phase III trials for GE cancer patients suggests that new targeted therapies, either alone or combined with inhibiting EGFR/RTK, should be considered. In this review, we intent to discuss an emerging new drug target, known as the store-operated Ca^2+^ entry (SOCE), which is a key intracellular Ca^2+^ signaling pathway in GE cancers. We will summarize the recent advances in understanding pathophysiological role of each component of the SOCE machinery in GE cancer cell proliferation, migration, and invasion, as well as stemness maintenance. Lastly, we will discuss efforts towards development of more specific and potent SOCE inhibitors, which appear to be a new set of chemotherapeutic drugs appearing at the horizon to provide either targeted therapy or adjuvant treatment to overcome drug resistant in GE cancers.

## Dysregulated Intracellular Ca^2+^ Signaling in Cancer Cells

Ca^2+^ is a vital second messenger in the cells and controls multiple cellular processes including cell growth, cell migration, cell death, cell cycle, autophagy and downstream genes transcription (Cui et al., 2017). Thus, it is not a surprise that Ca^2+^ homeostasis is a key factor in the tumor initiation, angiogenesis, progression and metastasis (Chen et al., 2011; Chen et al., 2013a; Bergmeier et al., 2013; Yang et al., 2013). The intracellular Ca^2+^ signaling is known as a complicated network composing of different Ca^2+^ movements, such as Ca^2+^ spikes (Baudouin-Legros et al., 2003), waves and oscillations (Ronde et al., 2000; Giannone et al., 2002; Lewis, 2003). They are spatially temporally orchestrated with many channels and transporters playing in the show. First, inositol 1, 4, 5-trisphosphate (IP_3_) receptor or ryanodine receptor (RyR) mediates Ca^2+^ stores such as endoplasmic/sarcoplasmic reticulum (ER/SR) (Mikoshiba et al., 1994). Secondly, ER/SR Ca^2+^-ATPase (SERCA) pumps Ca^2+^ from cytosol back to ER/SR. Plasma membrane (PM) Ca^2+^-ATPase drives Ca^2+^ from cytosol to extracellular space (Santulli and Marks, 2015). Thirdly, mitochondrial Ca^2+^ uniporter (MCU) controls mitochondrial Ca^2+^ uptakes. Fourthly, PM Ca^2+^ channels or transporters mediate Ca^2+^ influx from extracellular space into cytosol, which include voltage-gated Ca^2+^channel, transient receptor potential channel (TRP), SOCE channel, Na^+^/Ca^2+^ exchanger (NCX) and purinergic receptor, etc. Since these Ca^2+^ channels or transporters contain extracellular domains as good drug targets, they received great attention for chemotherapy researchers. Among them, SOCE channel has been actively investigated.

## Store-Operated Calcium Entry Machinery

SOCE was first reported about three decades ago in the name of capacitative Ca^2+^ entry (CCE) (Putney, 1986). In this pathway, activation of the G-protein coupled receptor (GPCR) leads to the stimulation of PLC to generate IP_3_, which in turn causes intracellular Ca^2+^ release, followed by a reduction of Ca^2+^ concentration inside the ER lumen. The reduced ER Ca^2+^ store sends a signal to the PM to activate CCE, allowing refill of the reduced ER Ca^2+^ store (Ma and Pan, 2003; Pan et al., 2014; Pan and Ma, 2015; Cui et al., 2017). This ER Ca^2+^ store-dependent Ca^2+^ influx is also known as Ca^2+^ release-activated Ca^2+^ (CRAC) current. The identification of SOCE machinery has been a long journey and hitherto, a consensus view is that SOCE machinery is complex with various tissue and cell specific components.

### Orai and STIM Molecules

The two main group members in SOCE machinery in mammalian cells are stromal-interacting molecule (STIM1 and STIM2) and Orai (Orai1, Orai2, and Orai3) molecules (Bergmeier et al., 2013). STIM1 and Orai1 were the first ones to be identified and have been well studied (Yang et al., 2009). STIM1 is an ER transmembrane protein with a luminal EF-hand which could detect changes in the ER Ca^2+^ content (Roos et al., 2005; Zhang et al., 2005; Luik et al., 2006; Mercer et al., 2006). Orai1 locates at PM and constitutes the pore forming unit of the SOCE channel (Yeromin et al., 2006). Upon ER Ca^2+^ store depletion in physiological or pathological cases, STIM1 molecules are active and translocate to the ER/PM junctional region (Ong et al., 2007). Then, they conjugate with Orai and, subsequently, activate to allow extracellular Ca^2+^ influx into the cytosol [39] (Ma and Pan, 2003; Huang et al., 2006; Soboloff et al., 2006; Spassova et al., 2006; Vig et al., 2006). Both Orai1 and STIM1 are ubiquitously expressed in almost all type of cells, including gastric and esophageal epithelial cells with important cellular functions. For example, SOCE mediated Ca^2+^ influx have been shown to be required for gastric epithelial renewal and repair (Kokoska et al., 1998; Engevik et al., 2020). Our studies demonstrated both STIM1 and Orai1 are expressed in esophageal epithelial cells and control cell proliferation (Zhu et al., 2014a; Choi et al., 2018) and eotaxin-3 secretion (Odiase et al., 2021).

### Transient Receptor Potential Canonical Molecules

Besides Orai and STIM molecules, transient receptor potential canonical (TRPC) family of Ca^2+^ permeable channels are also involved in the SOCE pathway. TRPC channels are tetrameric molecules with six transmembrane domains on each subunit and located on PM. In human cells, there are 6 TRPC channels divided into 2 families according to their biological functions: 1) TRPC1, TRPC4, TRPC5; 2) TRPC3, TRPC6, TRPC7 (Zhu et al., 1995). Their activation results from the stimuli induced PIP2 hydrolysis (Venkatachalam and Montell, 2007). TRPCs appear to interactive with other proteins, such as caveolin, junctate, and junctophilin (Ong and Ambudkar, 2015). The interaction with caveolin helps TRPC3 to be recruited to a channel complex within ER/PM junctions and form a functional signaling complex (Adebiyi et al., 2011). TRPCs may even indirectly interact with Orai in ER/PM complex with the requirement of STIM1 (Choi et al., 2014). While not all the TRPCs are involved in the SOCE signaling, TRPC1 and TRPC4 are clearly documented to mediate SOCE. TRPC1 was identified to be the first of the TRPC channels that was involved in SOCE in mammalian cells (Liu et al., 2003). It has been reported that TRPC1 mediates SOCE in secretory cells (Hong et al., 2011), vascular endothelial cells (Ma et al., 2011), smooth muscle cells (Dietrich et al., 2006), and endothelial cells (Tiruppathi et al., 2006). TRPC4-mediated SOCE is demonstrated in mouse mesangial cells (Wang et al., 2004), human adrenal cells (Philipp et al., 2000), both mouse and human endothelial cells (Sundivakkam et al., 2012), human gingival keratinocytes (Fatherazi et al., 2007), human corneal epithelial cells (Yang et al., 2005) and human pulmonary artery smooth muscle cells (Zhang et al., 2004). TRPC3 may mediate SOCE depending on cell type and expression level (Kim et al., 2009). TRPC6-mediated SOCE has been well studied in breast cancer cells (Guilbert et al., 2008; Jardin et al., 2018; Jardin et al., 2020).

### Store-Operated Calcium Entry in Gastroesophageal Cancers

Alteration in SOCE has been observed in many diseases. While genetic mutations in Orai1 or STIM1 were found in immune disorders, skeletal muscle myopathy and heart hypertrophy (Feske, 2011; Le Deist and Capiod, 2011; Verbsky and Chatila, 2011; Berna-Erro et al., 2012; Fuchs et al., 2012; Shaw and Feske, 2012), changes in expression and/or channel complex components are more commonly reported in various types of malignant, including GE cancers.

We previously reported upregulated expression of Orai1 in tumor tissues compared to that in adjacent non-tumorous tissues in ESCC patients (Zhu et al., 2014a). The high expression of Orai1 is associated with poor disease-free and overall survival rates. Both gene manipulation and pharmacologic studies demonstrated that elevated Orai1 results in hyperactivity of intracellular Ca^2+^ oscillations and, thus, controls rampant cell proliferation in ESCC cells. Interestingly, an essential trace mineral nutrient zinc is able to inhibit Orai1-mediated SOCE in ESCC cells, which has been linked to its cancer prevention function (Choi et al., 2018).

Enhanced SOCE and overexpression of Orai1 and STIM1 have been found in GC as well (Kokoska et al., 1998; Wong et al., 2017). STIM1 can promote gastric cancer progression (Xu et al., 2016) and silencing STIM1 inhibits cell proliferation via arrest of the cell cycle at the G0/G1 phase and increases cell apoptosis *in vitro* (Liu et al., 2015). From a study with more than 300 GC patients, Xia, *et al* reported that Orai1 and STIM1 expressions were higher in GC tissues compared with adjacent non-tumor tissues (Xia et al., 2016). Similar to the study in ESCC, they also found that higher Orai1 and/or STIM1 expression is associated with more advanced stages and poor prognosis. Moreover, Wong, *et al* showed that lipopolysaccharide (LPS) stimulates SOCE and results in the activation of downstream NF-κB signaling pathway. It is well known that LPS is an enriched component of the outer membrane of gram-negative bacteria *H. pylori*, which is a major risk factor for GC and triggers chronic inflammation responses (Zhang and Pan, 2020). SOCE-activated nuclear translocation of NF-κB then increases the transcription and expression of cyclooxygenase-2, a major inflammatory gene (Wang et al., 2017a). On the other hand, suppressing Orai1 and STIM1 expression by a Ca^2+^-binding protein S100A14 has been shown to induce cell differentiation and inhibit cell metastasis in GC (Zhu et al., 2017).

The role of TRPC-mediated SOCE in GE cancers has been studied best on TRPC6. Similar to Orai1, TRPC6 is also overexpressed in ESCC tumor tissues compared with normal esophageal tissues in terms of both mRNA and protein levels and its high expression is associated with poor prognosis (Zhang et al., 2013). Shi, *et al,* demonstrated that TRPC6 is a key factor to control G2 phase transition in tumorigenesis of EC (Shi et al., 2009). Due to the important role of TRPC6-mediated Ca^2+^ signaling, it is not surprising to see that the inhibition of TRPC6 leads to cell cycle arrest via Cdk1 in ESCC cells and decreased tumor formation in a mouse xenograft ESCC model (Ding et al., 2010; Zhang et al., 2013). In GC epithelial cells, the TRPC6 has been shown to be upregulated on protein and mRNA level and was responsible for regulation of the cell cycle, as the inhibition of TRPC6 resulted in cell cycle arrest in the G2/M phase and inhibited cell growth (Cai et al., 2009). Moreover, treatment of xenografted GC nude mice with a TRPC6 blocker resulted in the inhibition of the development of tumor. The TRPC6 may form a channel complex with TRPC1 and TRPC3 to fulfill their function, which was demonstrated in a study on TGF-β1-induced epithelial–mesenchymal transition (EMT) in GC cells (Ge et al., 2018). The authors showed that TRPC1/3/6 complex mediates Ca^2+^ influx and actives downstream the Ras/Raf1/ERK signaling pathway and the inhibition of TRPC1/3/6 impedes TGF-β1-induced EMT. Using a newly developed potent TRPC6 antagonist, a separate study also showed that inhibition of this Ca^2+^ channel suppresses proliferation of several GC cell lines as well as GC tumor growth in a xenograft model (Ding et al., 2018).

## Targeting Store-Operated Calcium Entry Channels for New Chemotherapy Drugs in Gastroesophageal Cancers

### Store-Operated Calcium Entry Inhibitors

Since SOCE-mediated Ca^2+^ signaling pathways are associated with several hallmarks of cancer, targeting SOCE turns out to be an active area in chemotherapy drug development area. As a result, many SOCE inhibitors have been reported to have anti-cancer potential. In Table 1, we present a summary of these SOCE inhibitors, which may not be comprehensive, due to the swift advancement of this field. The earliest SOCE blocker to be used is SKF-96365, which was shown to inhibit cancer cell migration and tumor metastasis in breast (Yang et al., 2009) and cervical cancers (Chen et al., 2011). It was also used in our early work in ESCC and was demonstrated to decrease Orai1-mediated SOCE and to reduce tumor growth *in vivo* (Zhu et al., 2014a). 2-APB is another effective SOCE inhibitor with low selectivity. For this purpose, it was first reported as antagonist for IP_3_ receptor with much higher IC50 (Yamashita et al., 2011). It can reduce cell proliferation and tumorigenesis in gastric cancer and colorectal cancer progression (Sakakura et al., 2003) (Wang et al., 2015). ML-9, an inhibitor for myosin light-chain kinase (MLCK) and STIM1 puncta, can promote cell death and autophagy in prostate cancer (Kondratskyi et al., 2014). RO2959, a novel, potent and selective SOCE inhibitor, inhibits gene expression, cytokine production, and proliferation in T cells (Chen et al., 2013a). SB01990, SPB06836, KM06293 and RH01882 are a cluster of SOCE inhibitors targeting and altering the Ca^2+^ selectivity filter of Orai1 (Sampath and Sankaranarayanan, 2016). GSK-5503A and GSK-7975A are Orai1-and Orai3-mediated SOCE inhibitors that slowly affect SOCE currents with no effect on STIM1–Orai1 coupling (Derler et al., 2013). Furthermore, the compounds also suppress TRPV6 channels, which is possibly because they share the target site (Jairaman and Prakriya, 2013). BTP2 or YM-58483 is a potent inhibitor for both CRAC and TRPC-mediated SOCE (He et al., 2005) (Yoshino et al., 2007). However, the mode of action may be more than direct channel antagonist. A report showed that BTP-2 can depolarize the cell membrane via TRPM4 activation and, thus contribute to the inhibition of SOCE and cytokine release (Takezawa et al., 2006). Carboxyamidotriazole, a non-selective SOCE inhibitor in non-excitable cell, has been approved to test its anti-tumor effects in Phase I and Phase II clinical trials for several cancers (Kohn et al., 2001) (Table 1). CM2489, CM3457 and CM4620 are three more selective SOCE inhibitors, which have been shown to prevent Ca^2+^ entry, and, thus, are used either to treat moderate-to-severe plaque psoriasis, or to reduce acute pancreatitis severity, or to inhibit lymphocytes and T cell-derived cytokine production (Ramos et al., 2012; Waldron et al., 2019) (Table 1). The Pyrazole analogs, including Pyr2, 3, 6 and 10, show different selectivity on TRPC3 and Orai1-mediated SOCE. Pyr10 is potent and selective for TRPC3-mediated SOCE. Pyr6 is potent to Orai1-mediated SOCE, while Pyr3 equally inhibits both channels (Schleifer et al., 2012).

**TABLE 1:** Store-operated calcium entry inhibitors.

Drug	Disease	Target	Clinical trial	References
SKF-96365	Breast cancer, cervical cancer, ESCC	TRPC, TRPV4, Orai1-STIM1		Yang et al. (2009), Chen et al. (2011)
2-APB	Gastric cancer, colorectal cancer	Orai1, TRPV2, IP3R1		Yamashita et al. (2011), Sakakura et al. (2003)
ML-9	Prostate cancer	STIM1 puncta		Kondratskyi et al. (2014)
RO2959	Inhibition on cytokine production and T cell proliferation	IP3-dependent CRAC		Chen et al. (2013b)
		Orai1		
SB01990, SPB06836, KM06293, RH01882	—	Orai1		Sampath and Sankaranarayanan (2016)
GSK-5503A, GSK-7975A	—	Orai1		Derler et al. (2013), Jairaman and Prakriya (2013)
BTP2/YM-58483	Antigen-induced airway inflammation	CRAC, TRPV4		He et al. (2005), Yoshino et al. (2007)
Carboxyamidotriazole	Ovarian cancer	CRAC	NCT00006486 (Phase 2, completed, metastatic kidney cancer)	Kohn et al. (2001)
			NCT00003249 (Phase 1, completed, advanced cancers)	
			NCT00003869 (Phase 3, completed, Stage III or IV non-small cell lung cancer)	
			NCT00019461 (Phase 2, completed, Fallopian Tube cancer, ovarian cancer, primary peritoneal cavity cancer)	
			NCT00019019 (Phase 1, completed, advanced solid tumors or refractory Lymphomas)	
			NCT00005045 (Phase 2, completed, advanced kidney cancer)	
			NCT00004146 (Phase 2, completed, newly diagnosed supratentorial glioblastoma)	
CM2489, CM3457, CM4620	plaque psoriasis, acute pancreatitis, sever COVID-19 pneumonia	CRAC	NCT04195347 (Phase1/2, recruiting, Acute Pancreatitis)	Ramos et al. (2012), Waldron et al. (2019)
			NCT03709342 (Phase 2, completed, acute pancreatitis)	
			NCT04661540 (Phase 2, recruiting, in critical COVID-19 pneumonia)	
			NCT04681066 (Phase 2, recruiting, acute pancreatitis and accompanying SIRS)	
			NCT03401190 (Phase 2, completed, acute pancreatitis and SIRS)	
			NCT04345614 (Phase 2, recruiting, severe COVID-19 pneumonia)	
Pyr2, 3, 6, and 10	Breast cancer	Orai1, TRPC3		Schleifer et al. (2012)
RP4010	ESCC and pancreatic ductal adenocarcinoma	Orai1	NCT03119467 (Phase 1, concluded, relapsed or refractory lymphomas)	Cui et al. (2018), Khan et al. (2020)

Among the above mentioned selective SOCE inhibitors, only two have been used in clinical trials but none for cancer treatment. CM2489 and CM 4620 from CalciMedica are used to block the production and release of pro-inflammatory cytokines from immune cells, and they are used in clinical trials for the treatment of plaque psoriasis and pancreatitis (Ramos et al., 2012; Waldron et al., 2019). During this pandemic, CM 4620 has also been evaluated in a new clinical trial for treatment of severe COVID-19 pneumonia (Table 1). With improved selectivity and reduced toxicity, modified forms of these SOCE channel inhibitors may still hold promise for further cancer therapeutic drug development. One of such compound will be discussed in great detail below.

### RP4010 in Gastroesophageal Cancer

RP4010 from Rhizen Pharmaceuticals is another selective SOCE channel inhibitor. Due to its low toxicity and high solubility, it has been successfully used in clinical trial in the form of an oral medicine. It was studied in a Phase I/Ib study for patients with relapsed or refractory non-Hodgkin’s lymphoma (NCT03119467). Additionally, its anti-cancer effects have been reported in both ESCC and pancreatic ductal adenocarcinoma (Cui et al., 2018; Khan et al., 2020).

Compared with other reported SOCE channel inhibitors, RP4010 is more potent in blocking SOCE in ESCC cells (Cui et al., 2018). In MTT assay, the IC50 of RP4010 is estimated about 1 µM for most tested ESCC cell lines whereas the IC_50_ of BTP-2 is 17 µM as best. Our studies showed that treatment of RP4010 resulted in reduction of intracellular Ca^2+^ oscillations, and caused cell cycle arrest at G0/G1 phase in several cultured human ESCC cell lines. This inhibitory effect on cell proliferation is caused due to decreasing nuclear translocation of nuclear factor kappa B (NF-κB). Moreover, it dramatically inhibits tumor growth in xenograft ESCC nude mice without observable adverse side effect, evidenced by normal histology results in all vital organs. Therefore, it remains to be a promising chemotherapy drug for GE cancers. Further, mechanistic study on the exact inhibitory effect on SOCE machinery and continue clinical evaluation are warranted.

## Targeting Store-Operated Calcium Entry in Gastroesophageal Cancer Drug Resistance

### Store-Operated Calcium Entry and Drug Resistance

Drug resistance is responsible for relapses of cancers and remains to be a big challenge in most cancer treatment. It includes resistance to classical chemotherapeutic agents or targeted therapies; it can occur at treatment (intrinsic) or is acquired after therapy. Accumulating evidence suggests that SOCE plays a significant role in drug resistance. The expression of Orai1 and STIM1 as well as SOCE are enhanced in therapy resistant ovary cancer cells (Schmidt et al., 2014). SOCE is required for the anti-cancer effect of cisplatin, a widely used conventional chemotherapy drug, in non-small cell lung carcinoma (Gualdani et al., 2019). Additional studies also showed that Orai1/STIM1-mediated SOCE is involved in 5-fluorouracil (5-FU), another widely used conventional chemotherapy drug, or gemcitabine resistance in pancreatic adenocarcinoma (Kondratska et al., 2014) and hepatocellular carcinoma cells (Tang et al., 2017). The proposed mechanisms, underlying the impact of SOCE on chemotherapy resistance, are attributed to inducing Ca^2+^ overload (Zheng, 2017), autophagy, EMT, activating MAPK and PI3K-Akt/Sgk signaling pathways (Wang et al., 2017a), upregulating transcription factors of NF-κB, c-myc, and p53 (Cui et al., 2017). Moreover, SOCE inhibitors decreased chemotherapy resistant cell migration in ovarian cancer (Huang et al., 2020).

### Ca^2+^ Signaling in Cancer Stem Cells

Cancer stem cells (CSC) are a subpopulation of tumor cells with capabilities of proliferating, self-renewing and differentiating. They are more resistant to chemotherapy drugs or radiation, which often leads to treatment failure and subsequent tumor recurrence (Hamburger and Salmon, 1977) (Bao et al., 2006; Li et al., 2008).

The identification and isolation of stem cells in GE tumors can be achieved by CSC markers using flow cytometry. Like adult stem cells, CSCs express the transcription factors SOX2, OCT-4, NANOG, and detoxification enzyme aldehyde dehydrogenase (ALDH) (Mohiuddin et al., 2020). While some cell surface markers, such as CD44, CD24, and CD133, have been identified as common CSC markers for almost all cancer types (Liu et al., 2006), CSC in GC and EC cancers present their specific markers as well (Takaishi et al., 2009). For example, Ming, *et al* showed that integrin α7 (ITGA7) is characterized as a functional CSC marker in ESCC (Ming et al., 2016). The known CSC markers in GE cancers are summarized in Table 2.

**TABLE 2:** Cancer stem cell markers in gastroesophageal cancers.

Cancer type	Markers
Gastric cancer	ALDH Nishikawa et al. (2013), CD44 Takaishi et al. (2009), CD44v8-10 Lau et al. (2014), CD133 Zhu et al. (2014b), CD24 Fujikuni et al. (2014), CD54 Chen et al. (2012), CD90 Xue et al. (2012), CD49f Fukamachi et al. (2013), CD71 Ohkuma et al. (2012), EpCAM Wenqi et al. (2009)
Esophageal cancer	ITGA7, CD44, ALDH, CD133, CD90 Wang et al. (2017b)

The stemness of CSC requires their distinct cellular characteristics, which is associated with different intracellular Ca^2+^ signaling compared with non-stem cancer cells (Cheng et al., 2018). Voltage gated Ca^2+^ channels (VOC) are a cluster of transmembrane proteins located on the cell membrane, which have been reported to regulate cell proliferation and migration in the cancer cells (Prevarskaya et al., 2018). VOC α2δ1 subunit (CACNA2D1) was identified as a CSC marker in diagnosis of hepatocellular carcinoma (Zhao et al., 2013). Data in small-cell lung cancer/non-small cell lung cancer indicates that VOC α2δ1 subunit increase the chemotherapy or radiotherapy resistance (Yu et al., 2018) that makes α2δ1 a target for treatment in the clinical setup. Moreover, t-type Ca^2+^ channel Caν3.2 is up-regulated and induces CSC proliferation in glioblastoma (Zhang et al., 2017). This also results in Cav3.2 being a potential target in cancer therapy.

For the ER resident Ca^2+^ channels, IP3R and RyR are required for CSC stemness and proliferation. In breast cancer, chemotherapy induced cytosolic Ca^2+^ release from ER via RyR1 causes enrichment of CSC *in vivo* (Lu et al., 2017). With RyR1 knockdown, the CSCs diminish in the severe combined immunodeficiency (SCID) mice model. IP3R induced- Ca^2+^ release is required for tumor growth and metastasis in melanoma (Marciel et al., 2018). Pharmacological inhibitors targeting Ca^2+^ release via IP3R, are employed to block CSC function in the treatment of glioblastoma (Dong et al., 2017).

### Store-Operated Calcium Entry in Cancer Stem Cells

**FIGURE 1:**
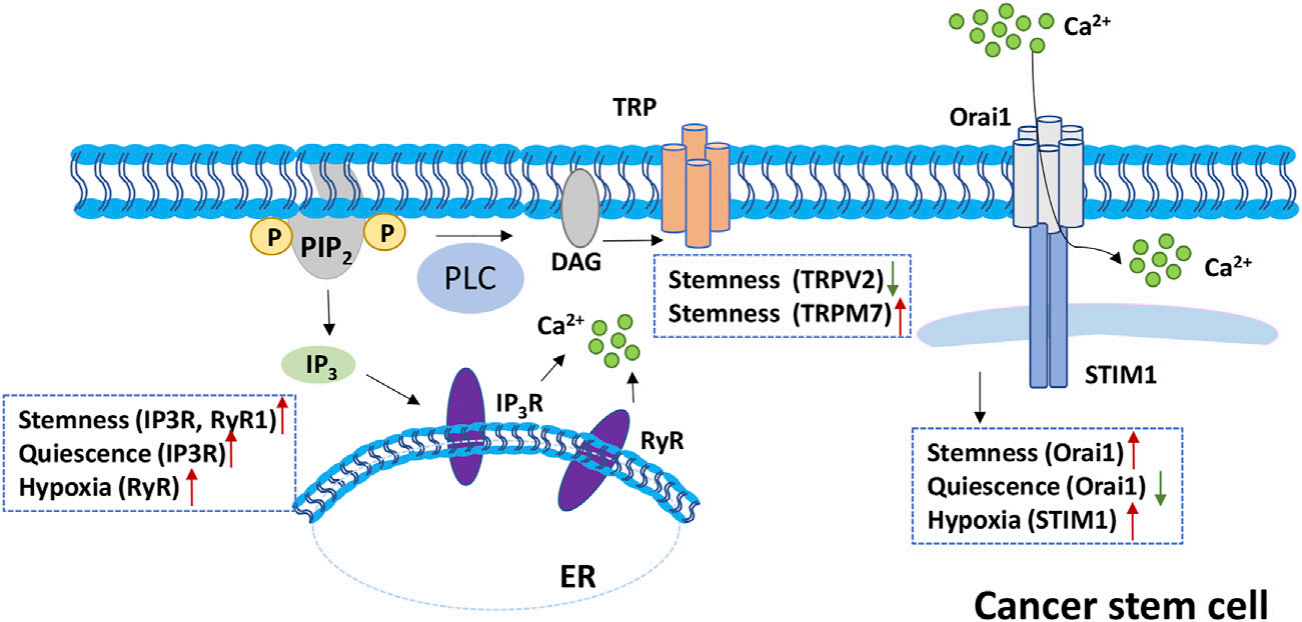
Role of SOCE in cancer stem cell (CSC). Left, IP3R and RyR are Ca^2+^ channels on the ER membrane. Activation of IP3R or RyR channels release Ca^2+^ from ER to cytoplasm. In CSC, IP3R and RyR promote stemness and quiescence. RyR also contributes to the cell hypoxia microenvironment. Middle, TRP family is involved in the stemness maintenance of CSC with negative and positive regulation of TRPV2 and TRPM7 separately. Right, Orai1 is upregulated in CSC assisting stemness maintenance; the downregulation of Orai1 promote quiescence maintenance. While STIM1 positively regulates CSC hypoxia microenvironment.

In addition to the VOC and IP3R/RyR, SOCE contributes to the stemness and differentiation of CSC as well (Figure 1). In oral/oropharyngeal squamous cells, Orai1 enhances cancer stemness by activation of NFAT pathway (Lee et al., 2016). By interaction with hypoxia-inducible factor-1 alpha (HIF-1α), STIM1 promotes the hypoxia-induced tumorigenesis in hepatocarcinoma (Li et al., 2015). Another study reveals that Orai1 mediated SOCE are essential for tumor invasion in glioblastoma (Motiani et al., 2013). Furthermore, the treatment of SKF-96365 can reduce CSC cell proliferation and decrease stemness in glioblastoma (Aulestia et al., 2018).

TRP channels may be involved in regulation of CSC function as well. Transient receptor potential melastatin 7 (TRPM7) enhances tumor migration and invasion by up-regulating expression of CSC, ALDH1, and CD133 in glioma cells (Liu et al., 2014). Transient receptor potential vanilloid 2 (TRPV2) is reported to inhibit CSC proliferation and promotes CSC differentiation in glioblastoma (Morelli et al., 2012). Knockdown of TRPV2 promotes colony formation and CSC expression in hepatocellular carcinoma (Hu et al., 2018).

In order to overcome cancer reoccurring issue, many clinical trials have been conducted focusing on elimination of CSC. Mithramycin, a selective inhibitor of transcription factor Sp1, is on phase 1 clinical trial for esophageal neoplasms with the identifier number of NCT01624090. Metformin, a widely used diabetes drug, has been administrated in combination with chemotherapy in several clinical trials to treat multiple cancers, including colon cancer (NCT01440127), ovarian, fallopian tube, and primary peritoneal cancer (NCT01579812). Both drugs may not show huge effects on diminishing the tumor size, but they significantly decrease the recurrence of tumor. Currently, there is no report on whether mithramycin or metformin alter the expression of SOCE, voltage-gated or other Ca^2+^ channels in gastroesophageal CSC. Regardless the answer, it could be a promising treatment to combine metformin with SOCE inhibitors for a superior anti-cancer effect with reduced drug resistance.

## Conclusion and Future Directions

In this review, we summarized the recent studies on the role of SOCE in GE cancers. Apparently, SOCE plays multiple important roles in cancer cells proliferation, migration, invasion, metastasis and stemness maintenance. EGFR-targeting therapy has been used in many other cancers but with limited benefits for GE cancer patients. Since Ca^2+^ plays a vital role in the EGFR signaling pathway, SOCE-mediated signaling pathway may crosstalk with EGFR pathway. We speculate that combined inhibitors for both SOCE and EGFR pathways could achieve better anti-cancer effects than single agent alone for GE cancer. While the function of SOCE in drug resistant and CSC in GE cancers remains unclear, it is reasonable to foresee that a similar association exist as that in other cancers. Compared with parental cancer cells, the drug resistance GE cancer cells and CSC may contain different SOCE components or ratio, which may present distinct SOCE channel properties. Targeting the SOCE channels, specific and potent SOCE blockers could be used as a new chemotherapy for GE cancers. Moreover, combining SOCE inhibitors with other chemotherapy drugs targeting both normal tumor cells and CSC may enhance treatment efficiency and prevent tumor re-occurrences. In the combined treatment, how to synergize the drugs and reduce the drug resistance? How to decrease the side effect of drugs and obtain a better prognosis? Further investigation is required along those lines.

## Abbreviations

ALDH, aldehyde dehydrogenase; CCE, capacitative Ca2+ entry; CRAC, Ca2+ release-activated Ca2+; CSC, cancer stem cells; CT, computerized tomography; EAC, esophageal adenocarcinoma; EC, esophageal cancer; EGFR, epidermal growth factor receptor; EMT, epithelial–mesenchymal transition; ER, endoplasmic reticulum; ESCC, esophageal squamous cell carcinoma; EUS, endoscopic ultrasound; 5-FU, 5-fluorouracil; GC, gastric carcinoma; GE, Gastroesophageal; HIF-1α, hypoxia-inducible factor-1 alpha; IP3, inositol 1,4,5-trisphosphate; ITGA7, Integrin α7; LPS, lipopolysaccharide; mAb, monoclonal antibody; MCU, mitochondrial Ca2+ uniporter; PET, positron emission tomography; PM, plasma membrane; RTK, receptor tyrosine kinases; SERCA, ER/SR Ca2+-ATPase; SOCE, store-operated calcium entry; SR, sarcoplasmic reticulum; TKI, tyrosine kinase inhibitor; TRP, transient receptor potential channel; vEGFR-2, vascular endothelial growth factor receptor-2
